# Propranolol and Weekly Paclitaxel in the Treatment of Metastatic Heart Angiosarcoma

**DOI:** 10.7759/cureus.12262

**Published:** 2020-12-24

**Authors:** Oraianthi Fiste, Apostolos Dimos, Vasiliki- Elpida Kardara, Konstantinos Ballasis, Athanasios Karampeazis

**Affiliations:** 1 Department of Medical Oncology, 401 General Military Hospital of Athens, Athens, GRC; 2 Department of Cardiology, 401 General Military Hospital of Athens, Athens, GRC; 3 Department of Oncology, 401 General Military Hospital of Athens, Athens, GRC

**Keywords:** heart angiosarcoma, cardiac neoplasms, propranolol, β-blockers

## Abstract

Heart angiosarcoma, the most frequent among cardiac malignancies, is an extremely rare vascular tumor known to carry a dismal prognosis. The spectrum of presenting symptoms depends on tumor’s size, its anatomic location, and its invasiveness, whereas imaging techniques including cardiac magnetic resonance are critical in the differential diagnosis between malignant and benign neoplasms. Despite there are various available systemic therapeutic regimens for advanced cardiac angiosarcomas, yet, it still remains unclear which of them offers the best survival outcome in general. We present the uncommon case of metastatic right atrium angiosarcoma in a young male patient, in which the combination of propranolol and weekly paclitaxel, as first-line treatment, showed promising activity with manageable toxicity. Given the existing strong rationale for repurposing propranolol in oncology, this therapeutic approach merits further investigation in prospective studies with heart angiosarcoma patients.

## Introduction

Cardiac angiosarcomas are exceedingly rare, fatal malignancies of endothelial origin, characterized by aggressive biological behavior and high metastatic potential, with a mean survival of 9.6-16.5 months [1]. They usually originate in the right atrial chamber and exhibit a strong preference for middle-aged male patients, who generally present with non-specific, insidious symptoms, including dyspnea, chest pain, fatigue, anemia, and weight loss; thus, providing a genuine challenge from a differential diagnostic standpoint [1]. Herein, we report an unusual case of metastatic right atrium angiosarcoma in a young male, who experienced a radiologic complete response in both cardiac ultrasound and computed tomography of chest and abdomen within three months after the initiation of propranolol combined with weekly paclitaxel.

## Case presentation

A 33-year-old, previously healthy, nonsmoking, Caucasian male presented with a mild, painless, bilateral lower extremity oedema and gradually worsening dyspnea on exertion of one-month duration. His electrocardiogram (ECG) demonstrated negative T-waves, chest radiography indicated right pleural effusion, whereas the transthoracic echocardiogram (TTE) revealed the presence of small pericardial effusion. High-sensitivity cardiac troponin, virologic, and immunologic testing were negative, whilst D-dimers level was high.

**Figure 1:**
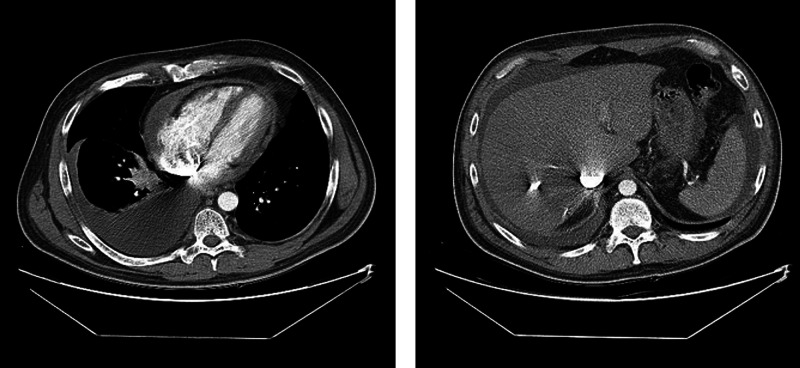
CTPA showing right pleural effusion with adjacent atelectasis, pericardial effusion, and mild ascites. CTPA: computed tomography pulmonary angiography.

**Figure 2:**
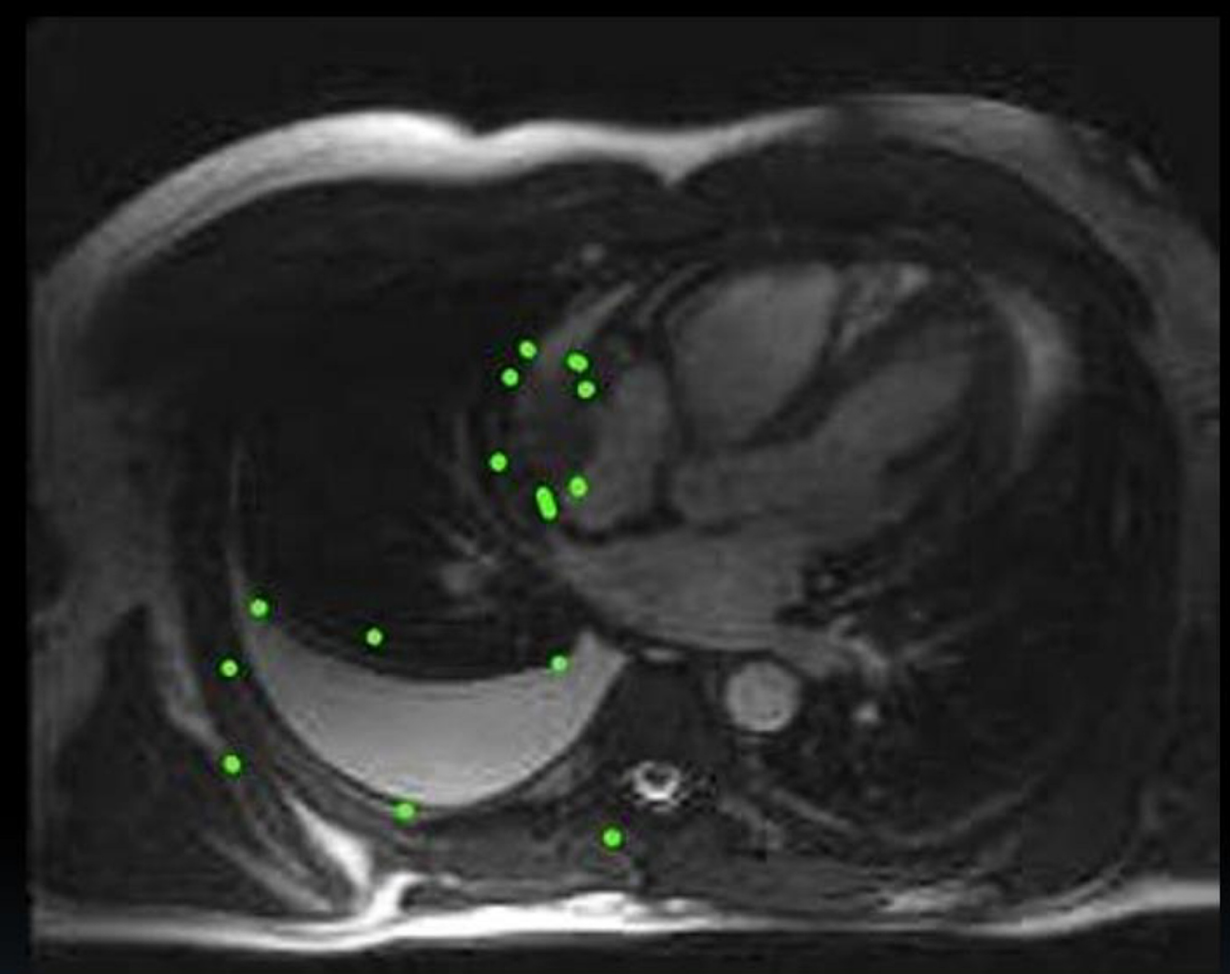
Cardiac MRΙ depicting a broad-based right atrial mass proximal to the inferior vena cava, which infiltrates the underlying myocardium and pericardium, with pericardial, and right pleural effusion.

A computed tomography pulmonary angiography (CTPA; Figure 1) excluded pulmonary embolism, yet confirmed the presence of both right pleural and pericardial effusion, together with mild ascites. A subsequent cardiac magnetic resonance imaging (CMR; Figure 2) depicted a broad-based right atrial mass that was infiltrating the underlying myocardium and pericardium. Finally, an endomyocardial biopsy via the right femoral vein, guided by two-dimensional transesophageal echocardiography, established the diagnosis of a primary heart angiosarcoma, whereas staging with positron emission tomography (PET) revealed widespread metastases in right scapula, in right first rib, and lungs.

Systemic combination treatment with weekly paclitaxel (150 mg/m^2^) and oral propranolol (40 mg three times daily) was commenced, resulting in prolonged disease control. Indeed, three months upon treatment initiation TTE revealed absorption of the pericardial effusion, whereas a computed tomography (CT) scan revealed substantial shrinkage of the pulmonary nodules. Second-line treatment with epirubicin plus ifosfamide was initiated eight months later, upon disease relapse in the liver, as it was revealed with routine follow-up scanning. The patient eventually died of multiple-organ progressive disease 17 months after the initial diagnosis.

## Discussion

Given its rarity, treatment guidelines for heart angiosarcoma have not yet been established. Often, the therapeutic approaches depend on published case reports and retrospective case series or extrapolations from the multidisciplinary approach of other soft tissue sarcomas [1]. A microscopically negative surgical margin (R0) resection may succeed in survival improvement, while the role of radiotherapy remains controversial, due to the potential cardiovascular complications including pericardial disease and cardiomyopathy [2]. Anthracycline-based chemotherapy is selected for patients with unresectable disease, whereas taxanes, ifosfamide, and gemcitabine have also shown antitumor activity [2]. Data from ALLIANCE and SARC028 trials suggest the promising effect of checkpoint inhibition [3,4], while anti-angiogenic targeted agents resulted in a modest response rate in several clinical studies [2]. 

Recently, the crucial role of adrenergic signaling in tumor growth and survival has brought β-blockers’ anti-proliferative and pro-apoptotic effects into sharp focus [5]. In a landmark study by Léauté-Labrèze in 2008, the nonselective inhibitor of β-1 and β-2 adrenergic receptors propranolol demonstrated significant efficacy in the treatment of benign infantile hemangioma [6]. In 2012 Chisholm et al. described the expression of adrenergic receptors on a variety of vascular tumors [7], while Stiles et al. exhibited the antitumor potential of β-adrenergic antagonists in a xenograft model of angiosarcoma [8].

Indeed, several preclinical studies have demonstrated the mechanisms by which β-blockers confer antitumor effects. Activated adrenergic receptors, by the neurotransmitters norepinephrine and epinephrine, (a) increase the number of regulatory T-cells (Tregs), (b) stimulate both the recruitment and differentiation of tumor-associated macrophages (TAMs), (c) suppress CD8+ cytotoxic T-cells, (d) promote both the migration and proliferation of endothelial cells, (e) generate the production of proangiogenic and inflammatory mediators, including IL-8, HIF-1α, IL-6, and VEGF [5]. Propranolol treatment inhibits these oncogenic processes, resulting in suppression of tumor cells’ proliferation and induction of tumor cells’ apoptosis [5]. 

Various retrospective studies and case reports translate the aforementioned findings from bench to the bedside, underlining the propitious outcomes of propranolol in aggressive vascular sarcomas [9-15]. In 2016 the European Medicines Agency (EMA) granted Orphan Drug Designation to propranolol against soft tissue sarcoma [16]. The optimal dose of propranolol for angiosarcoma remains to be determined.

Our patient had received 120 mg propranolol per day, divided into three doses, in combination with weekly paclitaxel, which resulted in progression-free survival (PFS) of eight months. Based on our current knowledge, paclitaxel monotherapy is a useful option for metastatic cardiac angiosarcomas, with a median PFS of four months [17]. Furthermore, in a multi-national retrospective analysis of 61 patients with primary cardiac sarcomas the initiation of first-line palliative chemotherapy resulted in a PFS of 4.4 months, whereas the median overall survival (OS) of patients with unresectable disease was 8.9 months [18]. Thus, our case highlights the potential efficacy of β-blockers in combination with paclitaxel for cardiac angiosarcoma’s therapeutic arena.

## Conclusions

Overall, our case underscores the promising activity of the combination of propranolol with paclitaxel-based chemotherapy for the treatment of metastatic heart angiosarcoma. Further prospective, randomized, controlled studies are warranted to further validate the efficacy of this economically sustainable treatment regimen for a highly lethal disease like heart angiosarcoma.
